# Determining the temporal, dose, and composition effects of nutritional substrates in an in vitro model of intrahepatocellular triglyceride accumulation

**DOI:** 10.14814/phy2.15463

**Published:** 2022-10-27

**Authors:** Shilpa R. Nagarajan, Eloise Cross, Elspeth Johnson, Fabio Sanna, Lorna J. Daniels, David W. Ray, Leanne Hodson

**Affiliations:** ^1^ Oxford Centre for Diabetes, Endocrinology and Metabolism, Radcliffe Department of Medicine Churchill Hospital, University of Oxford Oxford UK; ^2^ National Institute for Health Research Oxford Biomedical Research Centre Oxford University Hospital Trusts Oxford UK

**Keywords:** in vitro, liver, metabolism, saturated fatty acids

## Abstract

Pathological accumulation of intrahepatic triglyceride underpins the early stages of nonalcoholic fatty liver disease (NAFLD) and can progress to fibrosis, cirrhosis, and cancer of the liver. Studies in humans suggest that consumption of a diet enriched in saturated compared to unsaturated fatty acids (FAs), is more detrimental to liver fat accumulation and metabolism. However, the reasons for the divergence remain unclear and physiologically‐relevant cellular models are required. Therefore, the aims of this study were to investigate the effect of modifying media composition, concentration, and treatment frequency of sugars, FAs and insulin on intrahepatocellular triglyceride content and intracellular glucose, FA and circadian function. Huh7 cells were treated with 2% human serum and a combination of sugars and FAs (low fat low sugar [LFLS], high fat low sugar [HFLS], or high fat high sugar [HFHS]) enriched in either unsaturated (OPLA) or saturated (POLA) FAs for 2, 4, or 7 days with a daily or alternating treatment regime. Stable isotope tracers were utilized to investigate basal and/or insulin‐responsive changes in hepatocyte metabolism in response to different treatment regimes. Cell viability, media biochemistry, intracellular metabolism, and circadian biology were quantified. The FA composition of the media (OPLA vs. POLA) did not influence cell viability or intracellular triglyceride content in hepatocytes. In contrast, POLA‐treated cells had lower FA oxidation and media acetate, and with higher FA concentrations, displayed lower intracellular glycogen content and diminished insulin stimulation of glycogenesis, compared to OPLA‐treated cells. The addition of HFHS also had profound effects on circadian oscillation and gene expression. Cells treated daily with HFHS for at least 4 days resulted in a cellular model displaying characteristics of early stage NAFLD seen in humans. Repeated treatment for longer durations (≥7 days) may provide opportunities to investigate lipid and glucose metabolism in more severe stages of NAFLD.

## 1 INTRODUCTION

Nonalcoholic fatty liver disease (NAFLD) is the most prevalent chronic liver disease worldwide (Younossi et al., 2018) and is often, but not always, associated with insulin resistance (Cotter & Rinella, 2020). NAFLD covers a progressive spectrum of conditions that starts with the pathological accumulation of triglyceride (TG) in more than 5% of hepatocytes (Cotter & Rinella, 2020). The accumulation of intrahepatic TG (IHTG) represents an imbalance between fatty acid (FA) synthesis/storage and utilization/disposal. For example, individuals with higher IHTG (18%) are reported to have greater hepatic de novo lipogenesis (DNL) compared to individuals with lower IHTG (3%; Lambert et al., 2014).

As insulin is a key regulator of both systemic and hepatic FA and glucose metabolism, dysregulation in insulin concentrations (i.e., hyperinsulinemia) and/or signaling (i.e., insulin resistance) often coincides with pathological IHTG accumulation, although whether it is the cause or consequence remains unclear (Pramfalk et al., 2016). Greater IHTG has been suggested to alter hepatic glucose metabolism, such as glycogenesis and glucose production, however elucidating direct mechanisms in humans remains challenging. Lipid and glucose metabolism is also regulated by an internal molecular clock present in almost every cell of the body. This core circadian pacemaker oscillates within a 24‐h period and has been demonstrated to be affected by environmental cues, such as diet (Chaix et al., 2016); studies have reported rhythmicity is lost with increased IHTG accumulation.

Whole‐body in vivo studies in humans do not allow for the direct measurement of intracellular hepatic metabolism. In contrast, human in vitro studies, undertaken with either primary or immortalized cells, allow for the measurement of targeted outputs; however, robust and physiological in vitro hepatic models are lacking. While primary human hepatocytes are considered a gold standard, the high costs, limited culturing capability, potential for low cell viability, and the variability between donors and batches makes it challenging to use them as a workhorse model. Immortalized cell lines (e.g., Huh7 and HepG2) are commonly used as a more accessible and robust model; however, previous studies (Gómez‐Lechón et al., 2007; Penke et al., 2017; Su et al., 2019; Yan et al., 2019) have cultured hepatocyte cell lines in supraphysiological concentrations of nutrients (between 100 and 2000 μM of only one or two FAs [palmitate, oleate, or a combination of both], alongside 5.5–25 mM of glucose), which may, in part, explain why they have poorly recapitulated primary hepatocytes.

We previously described an in vitro model of IHTG accumulation, whereby manipulation of physiologically relevant concentrations of nutritional substrates led to a macrovesicular pattern of steatosis in Huh7 cells (Gunn et al., 2020). Our previous model used a more unsaturated FA (UFA) mix. However, human studies have shown that a diet enriched with saturated FAs (SFA) is associated with greater IHTG accumulation compared to a diet enriched with UFA (Yki‐Järvinen et al., 2021). Therefore, the aim of this study was to investigate the effects of altered media nutritional composition, concentration, and treatment frequency of nutrients and hormones to determine their influence on IHTG accumulation and intracellular glucose and FA metabolism and circadian function.

## 2 METHODS

### 2.1 Cell culture

Huh7 cells kindly provided by Dr. Camilla Pramfalk (Karolinska Institutet) were cultured in Dulbecco's modified Eagle's medium (DMEM) supplemented with GlutaMAX™, 5.5 mmol/L glucose, 1 mM sodium pyruvate, 2.4 g/L sodium bicarbonate (Gibco, Life Technologies), 10% fetal bovine serum (FBS, Seralab), 10,000 U/mL penicillin–streptomycin (P/S; Gibco, Life Technologies), and 1% nonessential amino acids (NEAA; Gibco, Life Technologies). Cells were maintained at 37°C in 5% carbon dioxide. All cells were regularly tested for mycoplasma contamination and only used for experiments if negative.

### 2.2 Experimental treatments

Cells were seeded into relevant plates at the following densities: 1 × 10^5^cells ml^−1^ (6‐well plate), 5 × 10^4^ cells ml^−1^ (96‐ and 24‐well plate) and cultured in maintenance media for 24 h prior to treating with experimental media. Experimental media consisted of glucose‐ and phenol‐free DMEM supplemented with 2% human serum (Lot# BRH1108782, Seralab), 1% P/S, 1% NEAA, 1% sodium pyruvate, 1% GlutaMAX™ (Gibco, Thermo Fisher), and a mixture of FAs (low fat [LF] = 200 μM FA mix; high fat [HF] = 800 μM FA mix) and sugars (low sugar [LS] = 5.5 mM glucose; high sugar [HS] = 11 mM glucose + 5.5 mM fructose) depending on the treatment conditions (i.e., LFLS, HFLS, HFHS). FA mixes were a combination of 4 FAs (oleic acid, palmitic acid, linoleic acid, and α‐linolenic acid) in one of two ratios: OPLA (45:30:24:1, respectively) and POLA (44:45:10:1, respectively). Overall, the difference between OPLA and POLA is primarily due to changes in the abundance of palmitate and linoleate. Insulin (Gibco, Thermo Fisher) and glucagon (Promocell) were also included to experimental media as described in the results. Duration and frequency of treatment consisted of either (i) 7 days of experimental treatment with media changed on alternate days (hereinafter called “alternate day treatment”) or (ii) 2 to 4 days of experimental treatment with media changed every day (hereinafter called “daily treatment”) following a 4‐day sensitization period in media containing human serum (Gunn et al., 2017, 2020).

### 2.3 Single cell gel electrophoresis (comet assay)

An assessment of DNA damage by single cell gel electrophoresis was carried out using the Comet SCGE assay kit (Enzo #ADI‐900‐166). Cells were cultured in treatment as previously outlined for 7 days. Assay was carried out according to the manufacturer's instructions following the alkali electrolysis method. DNA damage was quantified using OpenComet software (Gyori et al., 2014) on ImageJ.

### 2.4 Biochemical analysis

Glucose, lactate, TAG, apoB, and NEFA concentrations were measured in collected media on the AU480 chemistry analyzer (Beckman Coulter) and normalized to protein concentration (Gunn et al., 2017). Media ATP (Promega), acetate (Abcam) and intracellular glycogen (BioVision) were measured using plate‐reader assays according to manufacturer's instructions.

### 2.5 Determination of glucose output

After treatment, cells were washed three times in PBS before incubating in serum‐ and hormone‐free DMEM + 0.2% BSA for 16 h (overnight). The next day, cells were treated with Krebs‐HEPES buffer (0.12 M NaCl, 4.8 mM of KCl, 1.2 mM of MgSO_4_, 1.2 mM of KH_2_PO_4_, 2.5 mM of CaCl_2_, 150 mM of NaHCO_3_, 10 mM of HEPES) supplemented with 0.2% BSA, 1% NEAA, 2 mM of sodium pyruvate, 20 mM of sodium l‐lactate (Sigma Aldrich), insulin (0.1–100 nM), and glucagon (100 nM) for 20 min and harvested in 1% NP‐40 lysis buffer. Glucose output was measured in cell media using the Glucose‐Glo™ Assay from Promega according to manufacturer's instructions and normalized to protein concentration.

### 2.6 Lipid extraction and gas chromatography–mass spectrometry

Deuterium (10% v/v) was added to experimental media for the entire duration of the treatment (i.e., either 2, 4, or 7 days). Cells were harvested in 1% NP‐40 lysis buffer and lipids were extracted according to the Folch method (Folch et al., 1957). TG and phospholipid fractions were separated using solid phase extraction (Burdge et al., 2000) and methylated for measurement of fatty acid methyl esters (FAMEs) via gas chromatography (GC). A standard containing 31 known fatty acids was used to quantify FAMEs according to their retention times on a 6890N Network GC System (Agilent Technologies) and micromolar quantities were then summed to express each fatty acid as a percentage of this value (mol%). To measure intracellular de novo lipogenesis, incorporation of deuterium from ^2^H_2_O in the media into [^2^H]palmitate in intracellular TG and phospholipid fractions were measured by GC‐mass spectrometry (GC–MS) using a 5890 GC coupled to a 5973 N MSD (Agilent Technologies). Ions with mass‐to‐charge ratios (*m*/*z*) of 270 (M + 0) and 271 (*M* + 1) were determined by selected ion monitoring (Law et al., 2007).

### 2.7 Measurement of fatty acid oxidation

To trace the oxidation of exogenous FAs, unlabeled palmitate and oleate were replaced with D_31_‐palmitate and D_33_‐oleate in the experimental media for the duration of the treatment (i.e., either 2, 4, or 7 days) followed by media collection. The appearance of ^2^H_2_O in the media (derived from [D_31_]‐palmitate and [D_33_]‐oleate) using a Finnigan GasBench‐II (Thermo Fisher Scientific) was used as a marker of FA oxidation and calculated according to method of Law et al. (2007).

### 2.8 Luciferase activity assay

Huh7 cells (hepatocyte‐derived carcinoma cell line) were transduced with luciferase under the control of the PER2 gene which is a circadian promoter (PER2:Luc construct was kindly provided by Andrew Loudon, University of Manchester). Huh7 cells were seeded in 24 well plates and transduced with PER2::luciferase lentiviral vectors in low glucose (5 mM) DMEM containing 5 mg/mL Polybrene (Sigma; multiplicity of infection: 20). Following transduction, cells were plated in white 96 well plates and cultured in DMEM (Thermo Fisher Scientific) supplemented with 10% FBS, 1× penicillin/streptomycin, 1× lglutamine, and 5 mM of glucose. Twenty‐four hours after plating, cells were incubated in media containing either 0 or 800 μM of OPLA media supplemented with 2% human serum, 1× penicillin/streptomycin, 1× lglutamine, and 10 mM of glucose. Forty‐eight hours after plating, cells fresh media containing OPLA was replaced and 100 nM dexamethasone were added for synchronization. Cells were incubated in dexamethasone‐containing medium for 2 h in a 37‐degrees humidified and CO_2_‐buffered cell culture incubator. Following synchronization, cells were washed twice with HBSS, and fresh recording media (Thermo Fisher) containing 1× penicillin/streptomycin, 1× lglutamine, 10 mM glucose, and 1 mM luciferin was added. Real time PER2::luc recording was measured using BMG CLARIOstar Plus plate reader (BMG technologies) set at 37°C degrees and CO_2_‐buffered. Multicycle software (Actimetrics) was used for analysis, with data smoothed using adjacent‐averaging method, and 24‐h running baseline set.

### 2.9 Statistical analysis

All experiments consisted of at least three independent experiments, each with two or three technical replicates. Data are presented as mean ± SEM. Graphpad Prism was used for data analysis. One‐ and two‐way ANOVAs with Bonferroni's multiple comparisons tests were used to determine significant differences (*p* < 0.05).

## 3 RESULTS

### 3.1 Cell viability and DNA damage

**FIGURE 1:**
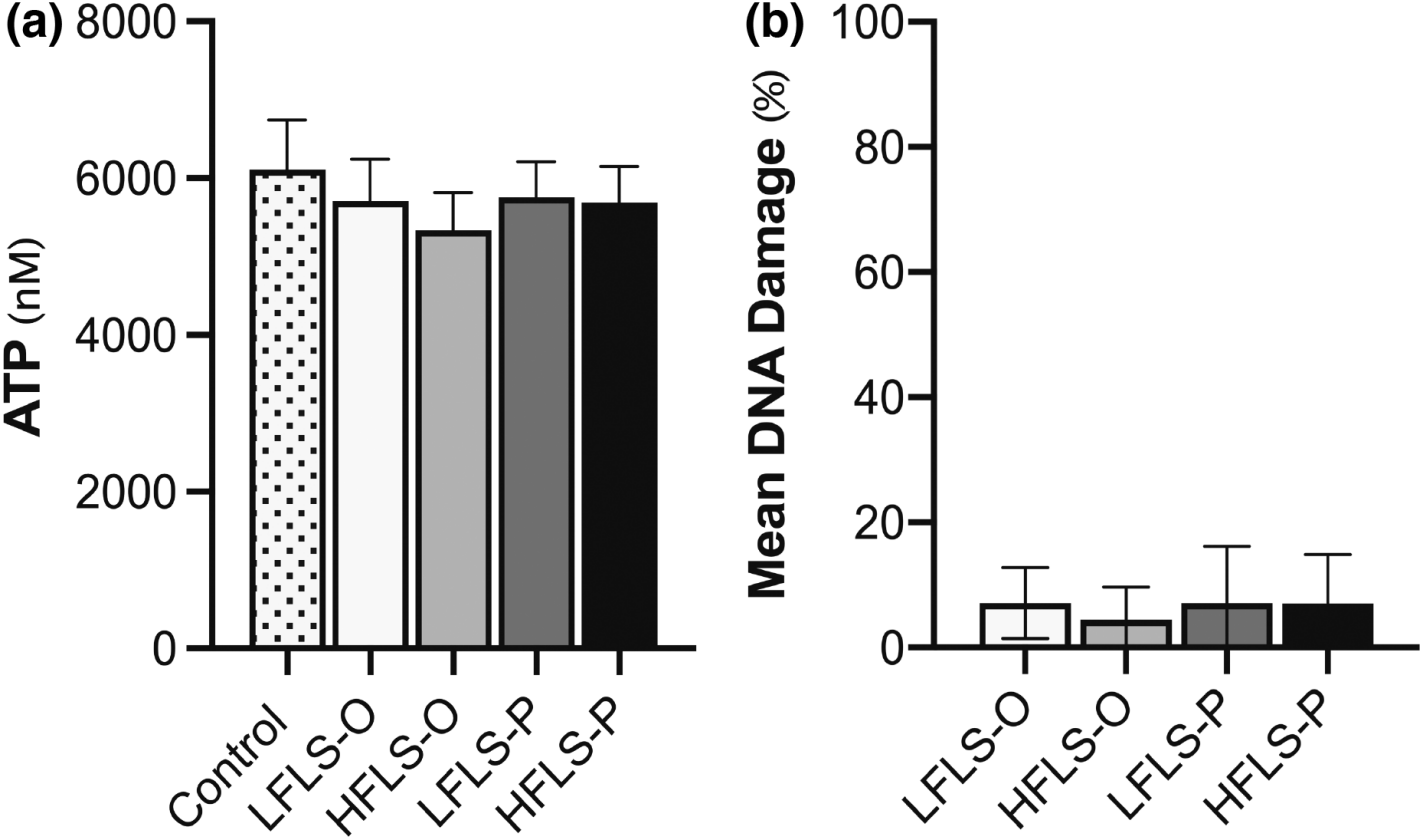
The effect of media concentration and composition on cell viability. Treatments consisted of either control (no fatty acids [FAs]; 5.5 mM glucose), low fat low sugar (LFLS; 200 μM FAs + 5.5 mM glucose) or high fat low sugar (HFLS; 800 μM FAs + 5.5 mM glucose) media, where FAs had a composition enriched in either unsaturated (O) or saturated (P) FAs. Cells were treated for 7 days with media changed on alternate days. (a) Intracellular ATP and (b) mean DNA damage were measured as markers of cell viability. All data are mean + SEM, *n* = 6 independent experiments, one‐way ANOVA with Tukey's multiple comparisons test.

Hepatocytes cultured in high amounts of palmitic acid have been previously reported as cytotoxic to cells (Chen et al., 2014; de Sousa et al., 2021; Penke et al., 2017). In this study, cells treated with either mixture of FAs (OPLA or POLA) on alternating days for 7 days displayed no difference in metabolic activity, as measured via cellular concentrations of ATP (Figure 1a), or DNA damage when measured by single‐cell gel electrophoresis (Figure 1b).

### 3.2 Intracellular fatty acid partitioning and TG secretion

**FIGURE 2:**
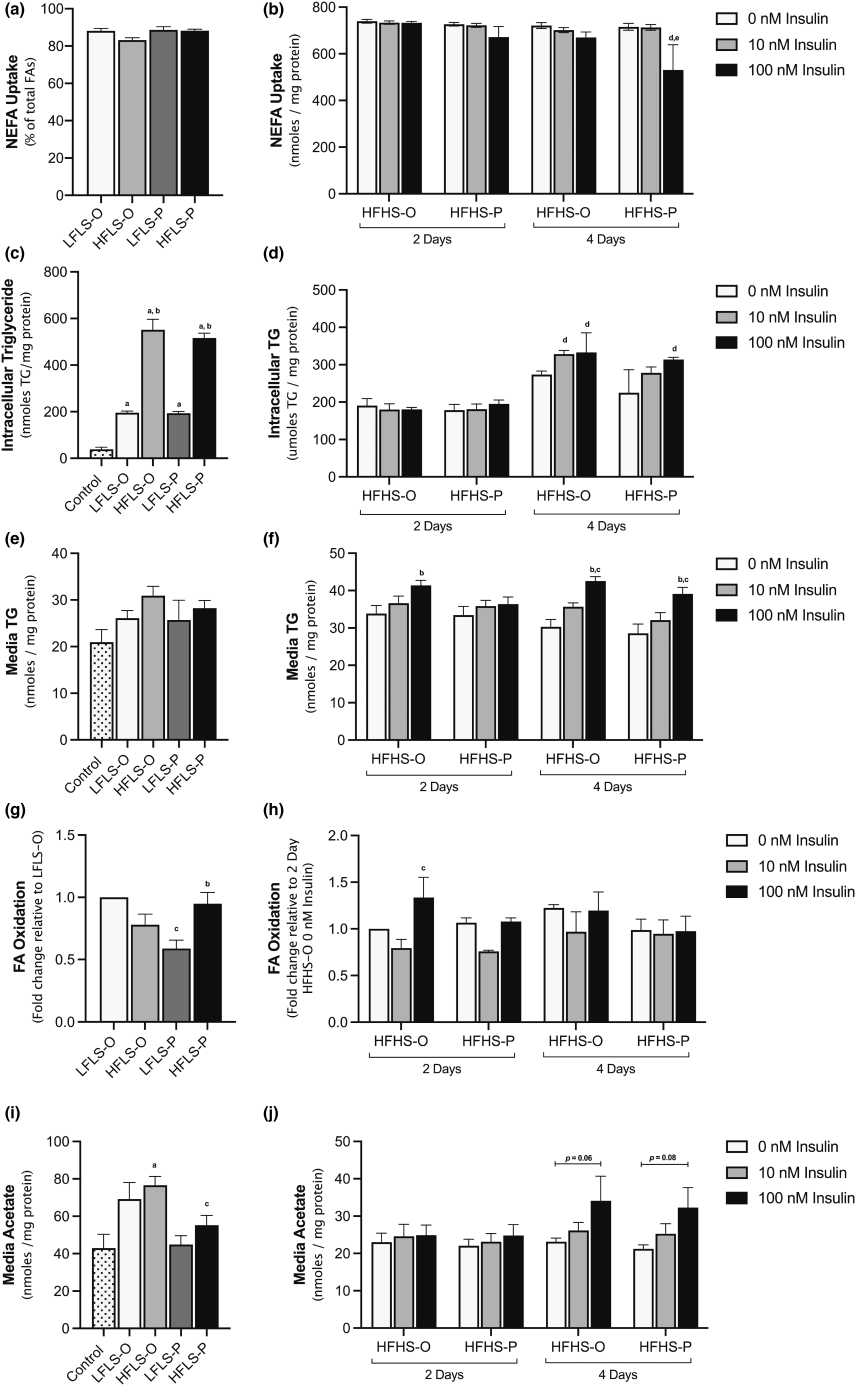
The effect of media concentration, composition, and frequency on hepatic fatty acid (FA) metabolism. Treatments consisted of either control (no FAs; 5.5 mM glucose), low fat low sugar (LFLS; 200 μM FAs + 5.5 mM glucose) or high fat low sugar (HFLS; 800 μM FAs + 5.5 mM glucose) media for cells treated for 7 days on alternate days, while cells treated daily for 2 or 4 days were treated with high fat high sugar (HFHS; 800 μM FAs + 11 mM glucose +5.5 mM fructose). FAs had a composition enriched in either unsaturated (O) or saturated (P) FAs. (a–b) Non‐esterified FA (NEFA) uptake from the media was calculated based on initial FA treatment concentrations. (c–d) Intracellular triglycerides (TG) were measured. (e–f) Media TG (g–h) media 2H2O enrichment and (i–j) media acetate were measured as markers of TG secretion, FA oxidation and peroxisomal oxidation, respectively. All data are mean + SEM corrected to protein concentration, *n* = 3–6 independent experiments, one‐way ANOVA or two‐way ANOVA with Tukey's multiple comparisons test.

Cells treated with OPLA or POLA on alternating days took up over 80% of extracellular FAs regardless of the concentration (200 vs. 800 μM) or composition (OPLA vs. POLA) of FAs (Figure 2a). To challenge the system further, cells that were treated with a high amount of FAs were also treated with a high amount of sugar (HFHS) and varying insulin doses (0–100 nM) daily for 2 or 4 days. FA uptake did not change with daily treatment of HFHS, except after 4 days of HFHS‐POLA and insulin (100 nM) treatment, where FA uptake significantly decreased (Figure 2b).

Once taken up, FAs can be esterified to form TG and stored in cytosolic lipid droplets. Intracellular TG storage was almost three times higher with 800 μM compared to 200 μM of FA treatment and this did not differ between the OPLA and POLA (Figure 2c). In daily treated cells, TG content increased by 50% after 4 days compared to 2 days of HFHS (Figure 2d). Although there was a trend toward higher TG content with insulin after 4 days of daily treatment, this was not significantly different (Figure 2d). Further, daily treatment with HFHS was associated with less TG content compared to alternate day treatment of LFLS or HFLS, even in the presence of insulin (Figure 2c,d). TG can also be secreted into the media in very low‐density lipoprotein (VLDL) particles. With alternate day treatment, exposure to 800 μM of FAs did not result in significantly more TG into the media compared to cells treated with 200 μM of FAs, regardless of FA composition (Figure 2e). Although insulin has been reported to inhibit VLDL secretion in humans and rodents (Chirieac et al., 2002; Lewis & Steiner, 1996), we found cells treated daily with HFHS had higher concentrations of media TG with 100 nM insulin; this becomes more pronounced after 4 days (Figure 2f). We measured media ApoB as a marker of VLDL particle number and found no difference between treatment groups (data not shown).

Intracellular FAs can be partitioned into oxidation pathways. Deuterium‐labeled FAs ([^2^H]‐FAs) were used to measure complete oxidation via the quantification of ^2^H_2_O production into the media (Law et al., 2007). Alternate day treatment with 200 μM of OPLA showed an almost 2‐fold higher FA oxidation than cells treated with 200 μM of POLA (Figure 2g). This divergence was not evident with 800 μM of FAs both with alternating and daily treatment (Figure 2g,h). Very long‐chain FAs undergo an additional peroxisomal oxidation step which can be measured via acetate production (Leighton et al., 1989). In cells treated on alternate days with POLA, there was approximately 33% less media acetate compared to OPLA‐treated cells (Figure 2i); this difference was lost with daily treatment of HFHS OPLA and POLA (Figure 2j).

### 3.3 Glucose uptake, storage, and oxidation

**FIGURE 3:**
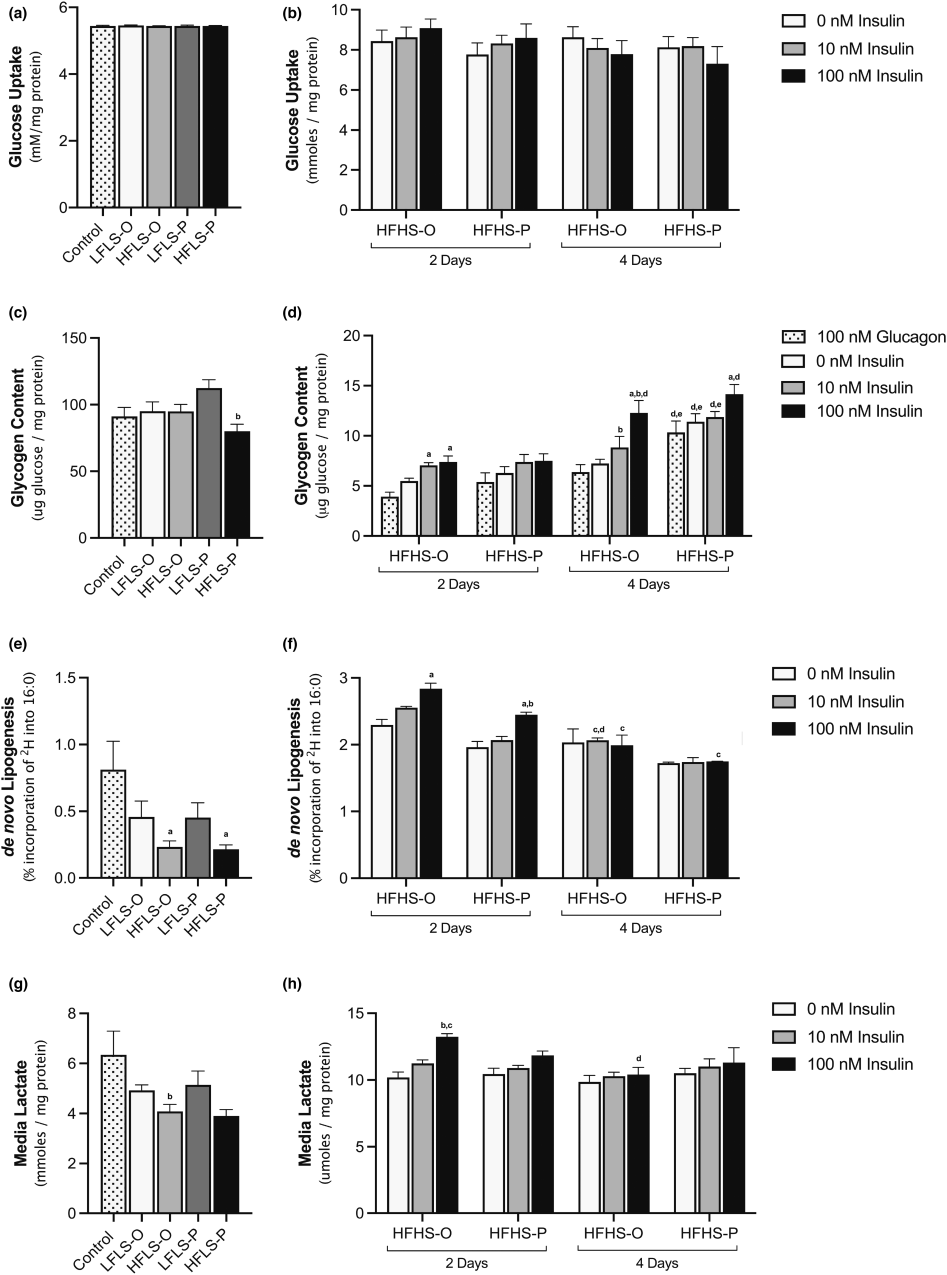
The effect of media concentration, composition, and frequency on hepatic glucose metabolism. Treatments consisted of either control (no fatty acids [FAs]; 5.5 mM glucose), low fat low sugar (LFLS; 200 μM FAs + 5.5 mM glucose) or high fat low sugar (HFLS; 800 μM FAs + 5.5 mM glucose) for cells treated for 7 days on alternate days, while cells treated daily for 2 or 4 days were treated with high fat high sugar (HFHS; 800 μM FAs + 11 mM glucose +5.5 mM fructose). FAs had a composition enriched in either unsaturated (O) or saturated (P) FAs. (a–b) Glucose uptake from the media was calculated based on initial glucose treatment concentrations. (c–d) Intracellular glycogen was measured. (e–f) De novo lipogenesis was calculated from deuterium appearance in C16:0. (g–h) Media lactate was measured as a marker of glycolysis. All data are mean + SEM corrected to protein concentration, *n* = 3–6 independent experiments, one‐way ANOVA or two‐way ANOVA with Tukey's multiple comparisons test.

In hepatocytes, glucose uptake is insulin‐independent. We assessed glucose uptake in both alternate (Figure 3a) and daily treatment (Figure 3b) from the media and found no difference with FA concentrations, compositions, or insulin dose, as expected. Glucose taken up into hepatocytes can be either stored as glycogen, oxidized via glycolysis, or converted into FA via DNL. Alternate day treatment with OPLA and POLA did not significantly alter intracellular glycogen concentrations compared to a no‐fat control (Figure 3c). However, there was a significant difference in glycogen concentrations between the 200 and 800 μM‐treated POLA cells (Figure 3c). The addition of insulin in combination with higher amounts of media sugars in the daily treated cells resulted in a differential response between OPLA and POLA. Cells exposed to 800 μM OPLA showed increased insulin‐stimulated glycogen content; however, cells given 800 μM of POLA did not produce a significant insulin‐dependent increase (Figure 3d). Notably, glycogen was significantly higher in all POLA‐containing treatments compared to equivalent OPLA treatments on day 4 (Figure 3d). Apart from glycogen, intracellular sugars can be stored as FAs via DNL. Cells treated with 800 μM of FAs on alternate days had lower DNL compared to cells treated with 200 μM, regardless of FA composition (Figure 3e). In daily treated cells, 100 nM of insulin increased DNL after 2 days but not after 4 days with both OPLA and POLA (Figure 3f). Further, the percent of newly synthesized palmitate is lower after 4 days of daily high‐fat treatment, likely representing increased TG content (Figure 3f).

Lactate secretion, a marker of intracellular glycolytic activity, has previously been shown to decrease with the addition of 800 μM compared to 200 μM of OPLA (Gunn et al., 2020). Consistent with this, we found cells treated on alternate days with 800 μM had a significantly lower media lactate concentration compared to cells treated with 200 μM of OPLA. Although the same trend was seen in cells treated with POLA, this was not significantly different (Figure 3g). After 2 days of daily treatment with HFHS‐OPLA and 100 nM of insulin, media lactate was significantly higher compared to a no‐insulin control; in POLA‐treated cells, there was no significant increase (Figure 3h). This insulin‐stimulated response disappeared after 4 days of daily treatment (Figure 3h).

### 3.4 Circadian rhythmicity and gene expression

**FIGURE 4:**
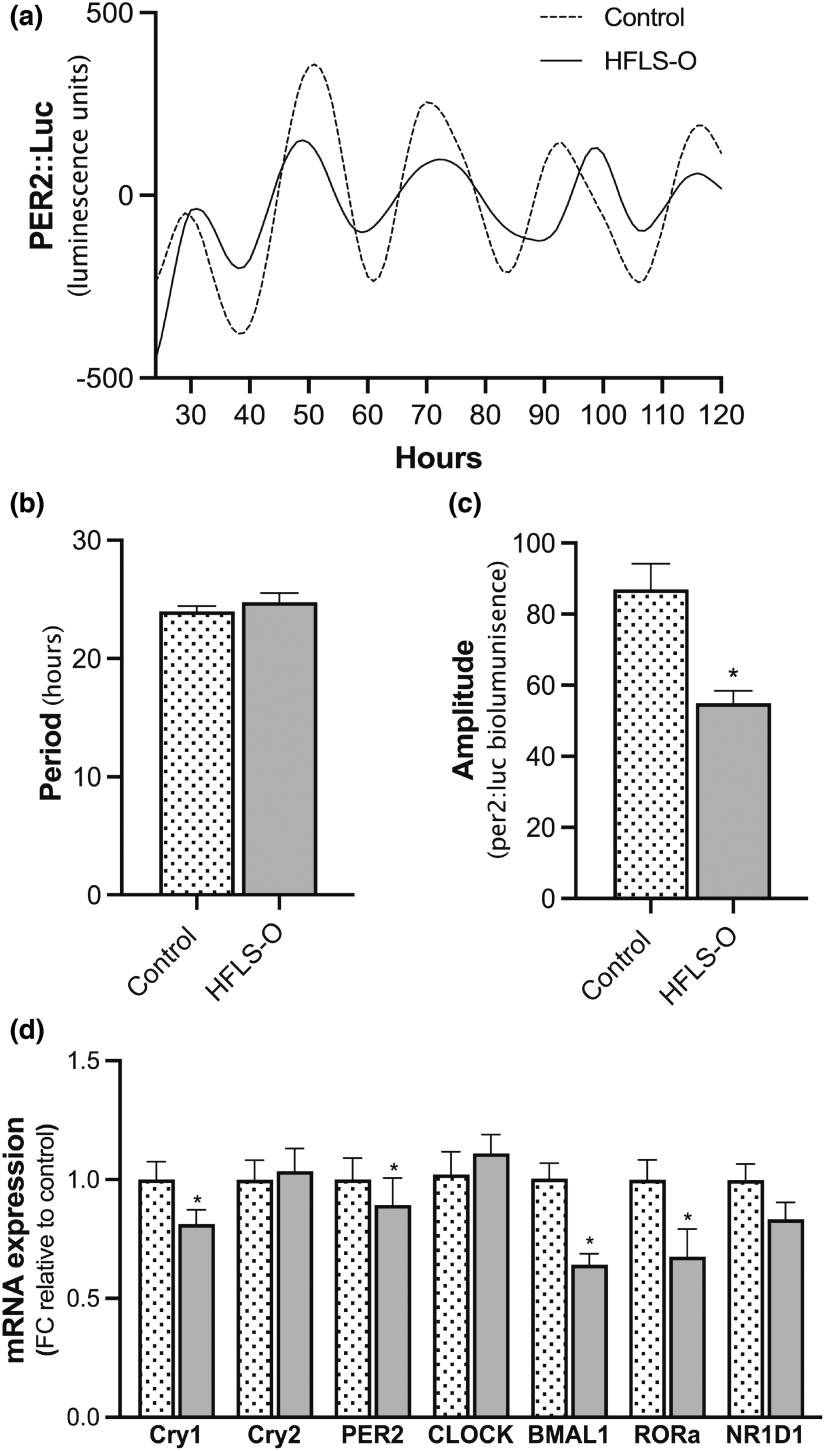
The effect of media composition on hepatic circadian function. Treatments consisted of either control (no fatty acids [FAs]; 5.5 mM glucose) or high fat low sugar (HFLS; 800 μM FAs enriched in unsaturated FAs (O) + 5.5 mM glucose). (a) Real time report per2‐luc drive bioluminescence after 48 h in treatment media. (b) Amplitude and (c) period length analyzed using multi‐cycle software with a running average of 24 h. (d) Expression of key circadian genes after 7 days in treatment media changed on alternating days. All data are mean + SEM, *n* = 2 independent experiments with 30–40 replicates in each experiment for panels a–c and 12–18 replicates in each experiment for panel d. *p < 0.05, un‐paired students *t*‐test, where data did not meet normal distribution statistical assumptions for parametric test, nonparametric test was used (Mann Whitney, 4 variables).

The effect of lipid loading using palmitate or oleate on hepatocyte circadian function and metabolism has previously been reported (Qi et al., 2018; Tong et al., 2015). Huh7 cells transduced with per2:luc lentiviral reporter and treated with 800 μM of OPLA for 48 h showed no effect on period length however amplitude was significantly reduced (Figure 4a–c). Gene expression of Cry1, Per2, BMAL1, and RORα all significantly decreased after 7 days of 800 μM OPLA treatment (Figure 4d), with no difference in Cry2, Clock, and NR1d1 gene expression.

## 4 DISCUSSION

The liver plays a central role in the homeostatic control of lipid and glucose levels which can fluctuate over the course of a day depending on the nutritional state (Rui, 2014). Insulin plays a key role in switching cellular metabolism between the state of energy storage and release. Within the liver, exogenous FAs mix with those synthesized intracellularly and can be partitioned into esterification pathways for storage (as TG in lipid droplets) or secretion (in VLDL particles), or into oxidation pathways (complete or ketogenesis; Hodson & Frayn, 2011). Similarly, intracellular glucose can be stored (as glycogen), oxidized (via glycolysis), or secreted into systemic circulation (via glycogenolysis or gluconeogenesis). The complexity of these pathways and their interactions are challenging to study in humans in vivo and thus there is a need for robust physiological in vitro models. Although our previous work utilized nutritional substrates at physiological concentrations to develop an in vitro model of IHTG accumulation (Hodson & Frayn, 2011), we did not investigate the effect of FA composition or insulin on lipid and glucose metabolism nor have we determined the effect of nutritional substrates on cell rhythmicity, which is suggested to be altered with increasing intracellular TG content (Li et al., 2021). Therefore, the aim of this study was to investigate the effects of altered composition, concentration, and treatment frequency of media nutrients and hormones on IHTG accumulation, intracellular glucose and FA metabolism. Overall, we found that while FA composition had little effect on cell viability and IHTG levels, it had significant effects on VLDL secretion, glycogen content, and circadian oscillations.

Human dietary intervention studies have previously reported that consumption of a diet enriched in SFA compared to UFA increased IHTG content to a greater extent (Bjermo et al., 2012; Luukkonen et al., 2018; Rosqvist et al., 2014, 2019). By culturing cells with OPLA and POLA, we observed similarities in NEFA uptake which is consistent with the findings of others (Mashek, 2013). In contrast to the human in vivo work, we found no divergence in intracellular TG content between the FA treatments when given at the same concentration. Moreover, our observations are not in agreement with the majority of previous in vitro studies where some (de Sousa et al., 2021; Eynaudi et al., 2021; Mei et al., 2011; Moravcová et al., 2015; Ricchi et al., 2009), but not all (Ishii et al., 2015; Malhi et al., 2006; Wang et al., 2013) have reported a significantly greater TG content with oleate compared to palmitate treatment. Typically, these studies have cultured HepG2 cells in the chosen FA in concentrations between 200 and 1000 μM for 6 to 24 h. As these studies have tended to use Oil Red O for lipid quantification, they have rarely normalized the data (for example, by cell number or protein) or provided information on cell health. Previous in vitro studies have suggested that treatment with SFAs (namely palmitic acid) compared to UFAs (namely oleic acid) is cytotoxic and can lead to reductions in cell viability and metabolic activity (Chen et al., 2014; de Sousa et al., 2021; Penke et al., 2017). Studies that demonstrate lower intracellular TG content with exclusive palmitate treatment, compared to oleate treatment, also showed parallel increases in apoptosis and ROS formation, and any differences in intracellular TG disappeared when treated with a mix of FAs at different molar ratios (Moravcová et al., 2015). In the current work, we show that modulating the composition of SFAs, monounsaturated FAs and polyunsaturated FAs (PUFA) in the media, regardless of dose and duration did not influence the viability and functional health of Huh7 hepatocytes when given as a mixture of FAs in physiological concentrations. Therefore, it is possible that in a non‐lipotoxic environment, TG formation may be similar between SFA‐enriched versus UFA‐enriched treatments in cellular models.

Although we did not see a difference in intrahepatocellular TG content with the different FA compositions, which is in contrast to the in vivo human data, it is plausible that the discrepancy can be explained by a variety of peripheral metabolic fates for FAs. For example, the human in vivo liver is exposed to not only FAs but also TG‐rich chylomicrons and VLDL remnants which were not present in our cell culture media. Additionally, SFA‐ compared to PUFA‐enriched diets are reported to increase adipose tissue lipolysis in humans (Yki‐Järvinen et al., 2021) which may increase plasma NEFA levels and contribute to greater hepatic NEFA uptake and TG storage. Further, intracellular partitioning of FAs into oxidation and VLDL secretion may differ depending on FA species. We have previously shown that postprandial whole‐body oxidation of linoleate is greater than palmitate (Parry et al., 2021) which may explain the differences observed in IHTG accumulation between PUFA‐ compared to SFA‐enriched diets in humans. Consistent with this, we report here significantly lower oxidation with POLA‐treated cells compared to OPLA. The higher observed FA oxidation with insulin treatment is contrary to expectation however, we have previously observed higher whole‐body oxidation rates in hyperinsulinaemic compared to normoninsulinaemic men (Hodson et al., 2010). Therefore, it would be of interest to measure differences in the intracellular partitioning and effects on metabolic pathways of specific FAs within the liver.

Insulin mediates a number of metabolic pathways to stimulate energy storage and suppress energy release, including the suppression of VLDL and glucose production. Although alterations in FA availability have been suggested to impair insulin signaling, insulin regulation of VLDL metabolism has not been previously reported in vitro. We have found insulin suppression of VLDL‐TG concentrations in the media with 200 μM of FAs (data not shown) however, insulin had the opposite effect on hepatocytes when treated with 800 μM of OPLA and POLA, respectively. In this study using Huh7 hepatocytes, media TG concentrations increased with increasing insulin concentrations, suggesting a lack of insulin suppression on TG secretion; this was especially pronounced after 4 days of daily treatment. The chronic effects of hyperinsulinemia and early stages of hepatic steatosis on VLDL production have been shown in humans and rodents (Steiner & Lewis, 1996). In nondiabetic, hyperinsulinaemic men with NAFLD, insulin suppression of VLDL‐TG secretion and particle size was lower compared to men without NAFLD (31.9% vs. 64.7%), suggesting that greater export of TG from the liver may be an early physiological response to compensate for hepatic lipid accumulation (Steiner & Lewis, 1996). Further, chronic but not acute hyperinsulinemia have been shown to accompany elevated VLDL production regardless of plasma NEFA levels (Steiner & Lewis, 1996). This is consistent with our results whereby HFHS‐treated cells incubated in 100 nM of insulin daily for 4 days secreted significantly more TG into the media compared to cells treated with no insulin. Notably, these cells did not secrete more TG after 4 days compared to 2 days, despite a doubling of intracellular TG, suggesting a plateau had been achieved. VLDL‐TG secretion has been reported to plateau in humans, once IHTG content reaches a certain point (usually ≥10%) in healthy nondiabetic individuals (Fabbrini et al., 2008). It is possible that if we had continued with longer term daily treatment on the cells with the FA mixtures this may have revealed a further exacerbation of a more severe NAFLD‐like phenotype.

Glucose is taken up by hepatocytes and converted to glucose‐6‐phosphate to maintain the diffusion of glucose down its concentration gradient (Rui, 2014). Once in the cell, glucose‐6‐phosphate can then act as a precursor for glycogen synthesis or undergo glycolysis to generate lactate (incomplete oxidation), ATP (complete oxidation), or FAs (DNL). Insulin is a key mediator of intracellular glucose partitioning and upregulates glycolytic, glycogenic, and DNL pathways in the postprandial state in healthy individuals (Rui, 2014). Although studies have reported on the positive effect of insulin stimulation on glycogen synthesis and in hepatocytes (Nagarajan et al., 2019), evidence is limited for the interaction between FA availability and glycogen deposition. We compared 200 and 800 μM of FAs on glycogen content after 7 days of alternate day treatments, and found POLA‐treated cells had decreased glycogen content with higher FA concentration, while OPLA‐treated cells showed no difference. As FA treatment did not influence glucose uptake, the observation that higher amounts of POLA resulted in decreased glycogen content may be due to impairment in either glycogen synthesis or breakdown; it would be of interest to determine the specific direction of effect. Others have reported that treating HepG2 cells with 0.25 mM of palmitate for 24 h was associated with 30% less glycogen content and decreased pAKT, pGSK, and GS activity, suggesting alterations in insulin signaling (Gao et al., 2010). We investigated the effect of FA treatment on insulin‐stimulated glycogen synthesis and found that after 4 days, POLA but not OPLA treatment was associated with diminished stimulation of insulin on glycogen content. This is consistent with an in vivo human study showing that in healthy, nonobese individuals, elevated plasma NEFA levels interfered with insulin suppression of glycogen breakdown (Boden et al., 2002). In contrast, in isolated liver perfusions, both palmitate and oleate impaired insulin signaling but had no effect on glycogen content (Anderwald et al., 2007). It would be of interest to investigate and compare hepatic glycogen content and metabolism in individuals with metabolic versus genetic NAFLD as they are reported to have notably different liver FA compositions (Luukkonen et al., 2016).

The influence of nutritional challenges to the circadian clock have been widely reported using high‐fat diet‐fed rodent models (Eckel‐Mahan et al., 2013). While a small number of studies have measured targeted circadian outputs in human hepatocyte cell lines (Qi et al., 2018; Tong et al., 2015), the circadian or rhythmic capabilities of these cultured cells remains uncertain. Previously, Hep1 and HepG2 hepatocytes treated with palmitate for 24 h have been shown to disrupt the BMAL1‐Clock complex (Tong et al., 2015) and dampen the rhythmicity of circadian genes across time (Qi et al., 2018). Here, we utilized a physiologically relevant FA mix and have shown reduced amplitude of circadian oscillations and expression of key circadian genes including Cry1, PER2, BMAL, and RORα indicating intracellular fat alters circadian function at the transcriptional level. These findings indicate that Huh7 cells can be used as a circadian model to further investigate the impact of nutritional challenges on hepatic circadian properties.

There are some limitations to this work. We used Huh7 cells, a well‐established human hepatocyte cell line that does not fully recapitulate in vivo liver function due to being hepatoma‐derived. While Huh7 cells are reported to show characteristics of tumor metabolism, such as rapid glucose consumption and elevated lipogenesis, we and others have found that culturing hepatoma cells in human serum instead of FBS can improve metabolic function to more closely recapitulate primary human hepatocytes (Gunn et al., 2017, 2020; Steenbergen et al., 2013). We have previously shown that culturing Huh7 cells in human serum lowers DNL to levels reported in human liver, and improved insulin sensitivity, VLDL secretion, and FA oxidation (Gunn et al., 2017). Here, we provide further evidence that Huh7 cells display circadian oscillations and express key circadian genes that can be modulated based on media composition. We also did not compare our results to primary human hepatocytes, which are often considered a gold standard in vitro model however, they present as a costly option with high inter‐donor variability and are frequently sourced from older donors with pre‐existing liver diseases and/or therapeutic pre‐treatment (Ruoß et al., 2020). Finally, we focused on modulating the composition of metabolic nutrients that are often completely absent from commonly used media (e.g., FAs, fructose); it would be interesting to investigate the influence of amino acid concentrations on liver metabolism (Lagziel et al., 2020).

## 5 CONCLUSION

We have demonstrated that Huh7 cells exposed to a physiological mix of fatty acids that are enriched in UFA, have intact glucose, and lipid pathways despite accruing intrahepatocellular TG. In contrast, we found when Huh7 cells were exposed to a physiological mix of fatty acids that are enriched in SFA there were profound alterations in glucose and lipid pathways and the presence of insulin further exacerbated these effects suggesting a change in intracellular insulin signaling cascade. Overall, our model displays several characteristics of early stage NAFLD in vivo and further manipulation of the nutritional and hormonal substrates, along with prolonged exposure will provide the opportunity to investigate effects of intrahepatocellular TG accumulation on both lipid and glucose pathways.

## AUTHOR CONTRIBUTIONS

Shilpa R. Nagarajan and Eloise Cross: study concept and design; acquisition of data; analysis and interpretation of data; drafting and revision of the manuscript, Elspeth Johnson: acquisition of data, Fabio Sanna: acquisition and interpretation of data, and revision of the manuscript, Lorna J. Daniels: acquisition of data; analysis and interpretation of data, and revision of the manuscript, David W. Ray: revision of the manuscript, Leanne Hodson: study concept and design; data interpretation, obtained funding; drafting and revision of the manuscript. All authors read and approved the final manuscript.

## FUNDING INFORMATION

This work was supported by a Novo Nordisk Postdoctoral Fellowship run in partnership with the University of Oxford (S.R.N. and L.J.D.); the British Heart Foundation (Fellowship FS/15/56/31645 and FS/SBSRF/21/31013 to L.H.), the Oxford BHF CRE (RE/18/3/34214), and the Biotechnology and Biological Sciences Research Council Institute Strategic Programme Food Innovation and Health (BB/R012512/1 and its constituent project BBS/E/F/000PR10347) to L.H.

## CONFLICT OF INTEREST

The authors declare no conflict of interest.

## ETHICAL STATEMENT

The manuscript contains no data or description of human patients or animals; all the described work has been undertaken using the Huh7 cell‐line.
