# Correction to Determining the temporal, dose, and composition effects of nutritional substrates in an in vitro model of intrahepatocellular triglyceride accumulation

**DOI:** 10.14814/phy2.70000

**Published:** 2024-08-18

Shilpa R Nagarajan, Eloise Cross, Elspeth Johnson, Fabio Sanna, Lorna J Daniels, David W Ray, Leanne Hodson. Determining the temporal, dose, and composition effects of nutritional substrates in an in vitro model of intrahepatocellular triglyceride accumulation. Physiol Rep. 2022 Oct;10(20):e15463. doi: 10.14814/phy2.15463.

In the published paper, the units in Figure 2D of μmoles TG/mg protein and 3H of μmoles /mg protein were incorrect. These should have read nmoles TG/ mg protein for Figure 2D and mmoles / mg protein for Figure 3H.

There was also an omission in the legends of Figures 2 and 3, as it was not stated what the symbols on above the bars represented for the statistical analysis comparison. For Figure 2, the legend should have had included: For figures a, c, e, g, and i: a = *p* < 0.05 compared to control; b = *p* < 0.05 compared to low fat (within same FA condition); and c = *p* < 0.05 compared to OPLA (within same FA concentration) and for figures b, d, f, h, and j: a = *p* < 0.05 compared to 100 nM glucagon (within same FA condition); b = *p* < 0.05 compared to 0 nM insulin (within same FA condition); c = *p* < 0.05 compared to 10 nM insulin (within same FA condition); d = *p* < 0.05 compared to 2 days (within same FA and hormone condition); and e = *p* < 0.05 compared to OPLA (within same hormone condition and day).

For Figure 3, the legend should have included the following: For figures a, c, e, and g: a = *p* < 0.05 compared to control; b = *p* < 0.05 compared to low fat (within same FA condition); and c = *p* < 0.05 compared to OPLA (within same FA concentration) and for figures b, d, f, and h: a = *p* < 0.05 compared to 100 nM glucagon (within same FA condition); b = *p* < 0.05 compared to 0 nM insulin (within same FA condition); c = *p* < 0.05 compared to 10 nM insulin (within same FA condition); d = *p* < 0.05 compared to 2 days (within same FA and hormone condition); and e = *p* < 0.05 compared to OPLA (within same hormone condition and day).

We apologize for these errors. The corrections do not affect the interpretation of the results.

